# Secnidazole for treatment of bacterial vaginosis: a systematic review

**DOI:** 10.1186/s12905-019-0822-2

**Published:** 2019-10-21

**Authors:** Mohamed A. Abd El Aziz, Foruzan Sharifipour, Parvin Abedi, Shayesteh Jahanfar, Helen Marie Judge

**Affiliations:** 1 Department of obstetrics and gynecology, Benha University Hospital, Benha University, Benha, Egypt; 2 Midwifery Department, Kermanshah University of Medical Sciences, Kermanshah, Iran; 3 Midwifery Department, Menopause Andropause Research Center, Ahvaz Jundishapur University of Medical Sciences, Ahvaz, Iran; 4 School of Health Sciences, Health Professions 2239, Central Michigan University, Michigan, USA; 5 School of Medicine, Atlantic University, Saint Lucia, West Indies USA

**Keywords:** Secnidazole, Metronidazole, Bacterial vaginosis, Systematic review, Ornidazole

## Abstract

**Background:**

Bacterial vaginosis (BV) is one of the common vaginal infections among childbearing women. The usual treatment for BV is metronidazole; hence 30% of women have recurrence within 60 to 90 days after treatment. There are some studies which assessed the effect of secnidazole on BV. The aim of this systematic review was to investigate the effectiveness of secnidazole for treatment of BV.

**Methods:**

The Cochrane Library, MEDLINE (PubMed), Scopus, and Web of Science (all databases from inception till October 28, 2018) were searched. Primary outcomes were clinical cure rate and microbiologic cure rate and the secondary outcomes were adverse events. Data was extracted from eligible studies by two review authors individually and analyzed by RevMan 5.3.

**Results:**

Our search found six trials involving 1528 participants. Treatment with 2 g secnidazole could significantly reduce the risk of BV in patients with three or less episodes of BV in the last year by OR: 7.54 (95% CI, 3.89–14.60, *p* < 0.00001) and in patients with four or more episodes of BV in the last year (OR: 4.74, 95% CI: 1.51–14.84, *p* = 0.0.008). Secnidazole (2 g) could significantly increase the microbiologic cure rate in women with 3 or less episodes of BV in the last year (OR: 7.63, 95% CI: 2.30–25.33, *p* = 0.0009) but not in the women with 4 or more episodes of BV in the last year (OR: 20.17, 95% CI: 1.06–382.45, *p* = 0.05). The clinical cure rate, microbiological effect and the therapeutic cure rate of 2 g secnidazole was significantly more than that of 1 g secnidazole.

The results showed that the clinical cure rate of 2 g secnidazole was not different from the following medications: metronidazole (500 mg bid for 5 days), secnidazole plus vaginal metronidazole, 2 g single dose of oral metronidazole and 2 g secnidazole plus vaginal ornidazole.

**Conclusion:**

This review showed that 2 g and 1 g secnidazole were better than placebo, however, 2 g secnidazole was more effective than 1 g. Secnidazole 2 g was not different from metronidazole (500 mg bid for 5 days), or from secnidazole plus vaginal metronidazole, or 2 g single dose of oral metronidazole or from 2 g secnidazole plus vaginal ornidazole.

## Background

Bacterial vaginosis (BV) is a type of vaginitis that results from change in bacterial vaginal flora due to the loss of hydrogen-peroxide generating lactobacilli and excessive growth of anaerobic bacteria and Gardnella vaginalis. BV is the most common gynecologic infection in women of childbearing age, affecting 40–50% of women within this age worldwide [1]. The vaginal flora contains various lactobacilli species that help maintain vaginal acidity (pH < 4.5) [2]. Vaginal microbiology is determined by factors that affect the strength of bacteria to survive, including vaginal pH, the presence of lactic acid produced by lactobacilli and hormonal agents such as estrogen, which fills the epithelial cells of the vagina with glycogen and is converted to lactic acid by lactobacilli [3]. The incidence of BV in the United States is 29.2%, which affects approximately 21 million women, and this disorder is the leading cause of 10 million annual visits due to the vaginitis [4]. BV’s clinical symptoms are characterized by increased discharge which smells like fish and is uniform, dilute and grayish in color [5]. The known risk factors for this type of vaginitis include poor socioeconomic status, poor health level, early sexual activity, multiple sexual partners, psychological stress, and biogenetic factors [6]. Based on clinical criteria, the clinical diagnosis of BV is confirmed if there are at least three criteria. These criteria include: 1. vaginal discharge that is uniform and homogeneous, and is gray or white to yellowish in color, 2. PH of vaginal discharge equal or greater than 4.5. 3. positive whiff test (amine odor after adding 10% potassium hydroxide to vaginal discharge) and the presence of clue cells in the vaginal mucus smear sample (at least 1 in 5 cells of the vaginal epithelium) [7].

### Treatment of bacterial vaginosis

Failure to properly treat bacterial vaginosis can lead to serious complications such as postpartum endometritis, pelvic inflammatory disease, premature rupture of the fetal membranes, preterm delivery, increased risk of post-hysterectomy infection, chorioamnionitis, spontaneous abortion, recurrent urinary tract infection and increased risk of intraepithelial cervical neoplasia [8, 9].

Other complications of this infection include increased risk of sexually transmitted infections such as chlamydia, gonorrhea, HIV, and herpes simplex type 2, [10] so early diagnosis of BV and its treatment are important.

Usual treatments for BV include oral metronidazole 500 mg bid for 7 days, vaginal metronidazole gel 0.75% per day for 5 days, and vaginal clindamycin 2% per day for 7 days [11]. In spite of access to these regimens, there is 30% recurrence 60 to 90 days after treatment, which increases over time (50% in 12 months) [12]. Other treatments include actinazole from the nitroimidazole group [13] with a longer half-life than metronidazole (approximately 17 h compared to 8 h) for the treatment of vaginosis and strains of trichomoniasis are in usage in Asia and Europe [14]. In laboratory studies, the antimicrobial properties of acetyazol have been shown against many bacterial species involved in vaginosis.

Various clinical trials have reported the effects of one or two grams of oral secnidazole on the improvement of BV [15–18]. Despite the numerous clinical trials conducted in this area, there is no review study that compares the therapeutic effects of secnidazole with other treatments regarding the improvement of symptoms of BV. Therefore, the aim of the present systematic review is to examine the evidence from randomized clinical trials on the therapeutic effects of secnidazole on BV and compare it with metronidazole or placebo.

## Methods

### Types of studies

We recruited Randomized controlled trials (RCTs).

### Types of participants

Women (of all ages) diagnosed with bacterial vaginosis using Amsel criteria were recruited for this study.

### Types of interventions

Eligible trials compared single or combination treatment regimen (secnidazole) compared with conventional treatment (metronidazole) or placebo for bacterial vaginosis. We had no restriction regarding route of administration, dose, frequency or duration.

#### Types of outcomes

##### Primary outcomes

1. Clinical cure rate.
2. Microbiologic cure rate.
3. Therapeutic cure rate

##### Secondary outcomes

Adverse events such as yeast infection, valvovaginal pruritius, nausea, or increase liver enzymes.

We followed the Cochrane Collaboration reviewed methods for collection and analysis summary data. There was no limitation regarding to publication status, country, duration of follow-up or language. The search terms are presented in Additional file 1. We conducted the search on Cochrane Library (CENTRAL 2018), MEDLINE (PubMed), Scopus, and Web of Science (all databases from inception till October 28, 2018).

Two reviewers (FS, HJ) independently examined title/abstracts of all studies according to our inclusion criteria. One of the review authors (MAA) checked for discrepancies which were resolved by discussion. One review author (MAA) entered the data into Review Manager 5.3. A second author independently checked the data (FS). Using a pre-designed data extraction sheet, two reviewers (MAA, FS) independently extracted the data from the included studies. The relevant data extracted from the included studies were study details (dates when the research was conducted, geographic location, participant inclusion criteria, funding sources, publication date), participant characteristics, interventions details (type, duration, route of administration, dose), outcome details (type of outcome, outcome assessment method), and bias assessment details (data necessary to assess the risk of bias, as described below).

##### Risk of bias assessment

**Fig. 1:**
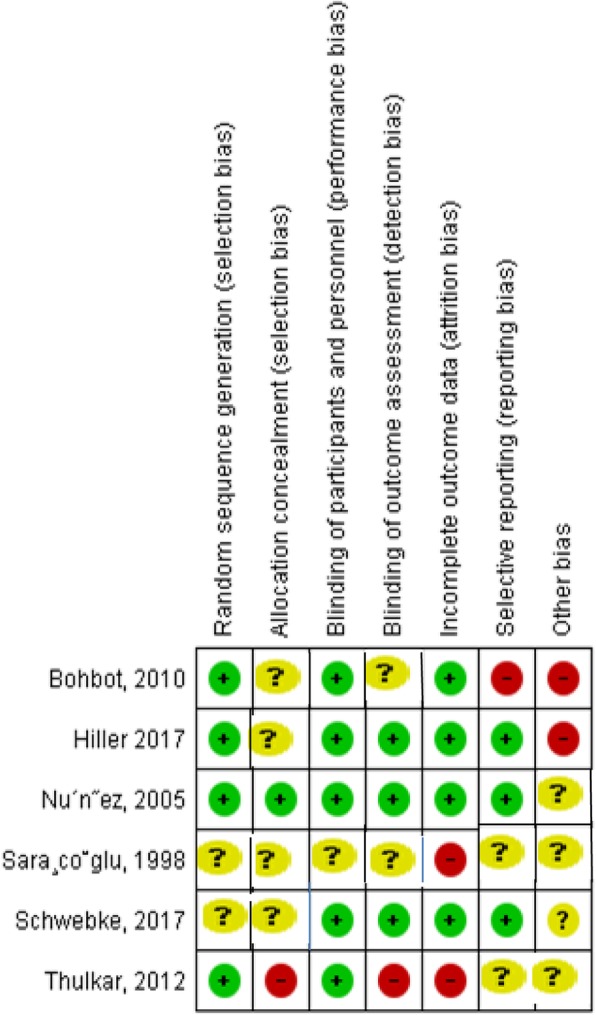
Risk of bias summary of included studies

We assessed the following risk of bias domains for each trial: random sequence generation (selection bias), allocation concealment (selection bias), blinding of participants and personnel (performance bias), blinding of outcome assessment (detection bias), incomplete outcome data (attrition bias), selective reporting (reporting bias), and other bias as shown in Fig. 1 [19].

#### Measures of treatment effect

All our outcomes were binary outcomes, so we calculated the odds ratio (OR) with 95% confidence intervals (CI). The unit of analysis was the participant.

##### Meta-analysis

Meta-analysis was only feasible for particular topics that at least two or more than two studies addressed those topics.

## Results

**Fig. 2:**
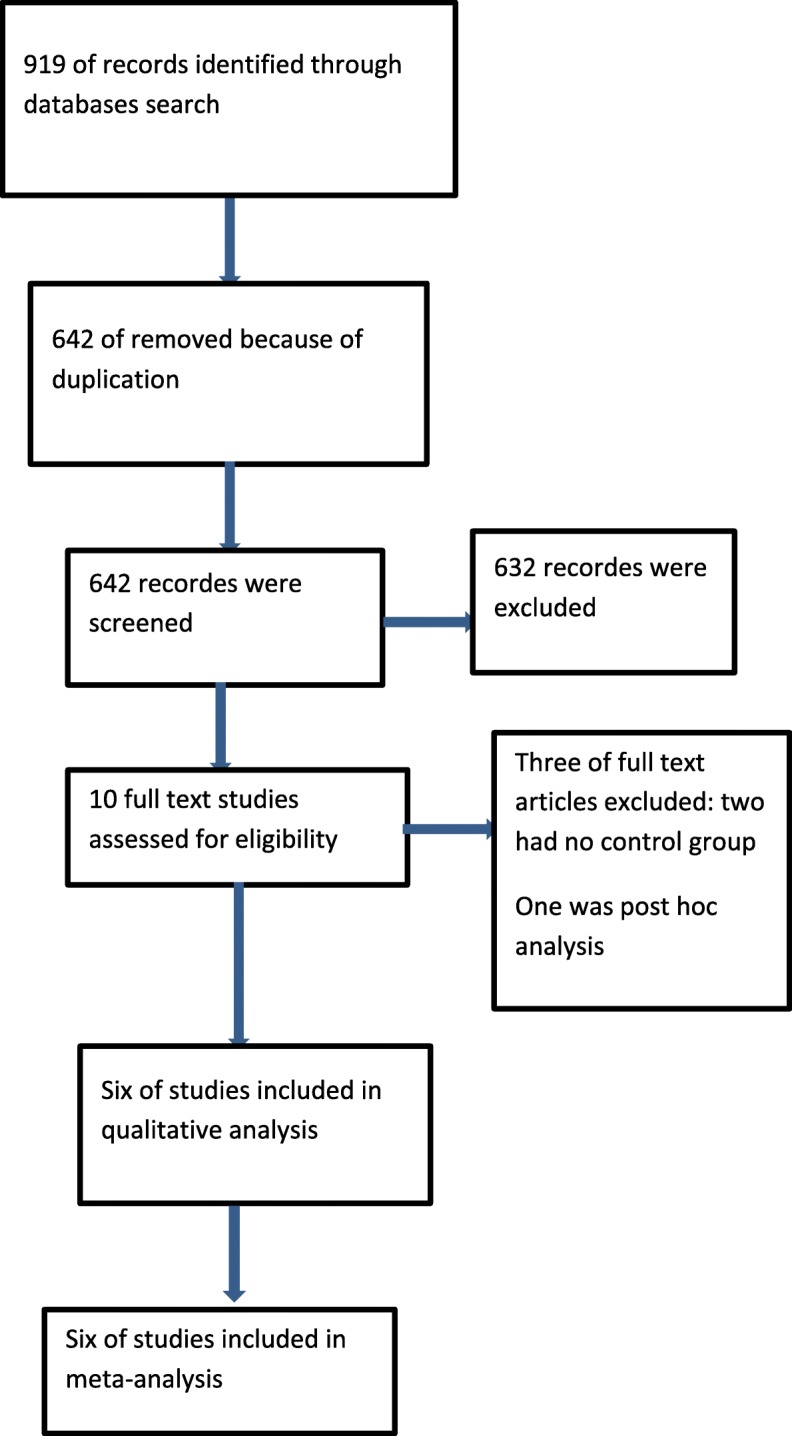
PRISMA chart

Our search strategies found six trials involving 1528 randomized participants that met our inclusion criteria (PRISMA Chart, Fig. 2) [13, 16, 17, 20–22].

The included trials were conducted between 1996 and 2014. Two out of our six studies have conducted in the USA. The remaining studies were conducted in France, Venezuela, Turkey and India. One trial was phase II, five were phase III (Table 1).

**Table 1:** Characteristics of included trials

Reference	Methods	Participants	Interventions	Outcomes	Notes
Hillier, 2017 [17]	- Parallel design - three arms - Phase II - Country: USA (24 ambulatory gynecology clinics) no single site contributed more than 16% to the study population . - Follow-up period: Efficacy was evaluated at 21–30 days post treatment. - Unit of randomization: participant - Analysis unit: participant	- Enrolled: 215 - Randomized: Secnidazole 1 g: 71 patients, Secnidazole 2 g: 72 patients, and placebo: 72 patient - Age: Median (min,max): Secnidazole 2 g: 31 (19, 54); Senidazole 1 g: 34 (19, 49), Placebo: 33 (19, 49) - Number of BV episodes in past 12 months (median(min, max): Secnidazole 2 g: 2 (1, 12); Senidazole 1 g: 1 (1, 13), Placebo: 3 (1, 12) - Number of BV episodes in past 12 months, n (%): 1) ≤3: Secnidazole 2 g: 41 (66.1); Senidazole 1 g: 44 (68.8), Placebo: 43 (69.4). 2) ≥4: Secnidazole 2 g: 21 (33.9); Senidazole 1 g: 20 (31.3); Placebo: 19 (30.6) - Baseline nugent score: median (min, max): Secnidazole 2 g:8 (4, 10); Senidazole 1 g: 9 (5, 10), Placebo: 8 (4, 10)	1. Secnidazole 2 g single oral dose 2. Secnidazole 1 g single oral dose 3. Placebo	- Primary: Clinical cure: based on the 1998 FDA guidance regarding evaluation of treatment for bacterial vaginosis: 1) Normal vaginal discharge, 2) negative 10% potassium hydroxide whiff test, and 3) clue cells less than 20% of total epithelial cells on microscopic examination of the vaginal wet mount using saline at the test of cure visit. - Secondary: 1) Nugent score (microbiologic cure): with a score of 0–3 considered Lactobacillus-dominant and a score of 4 or greater considered abnormal. 2) Therapeutic cure: defined as meeting the criteria for both clinical and microbiologic cure. - Safety: were based on the incidence, intensity, and type of adverse events and changes in patients’ physical examination findings, vital signs, and clinical laboratory results,	- Trial registration number: Clinical Trials.gov, NCT02147899 - A priori sample size estimation: yes - Trial conduction date: between May 28 and September 5, 2014 - Funding for this study was provided to Magee-Womens Research Institute (Hillier), Drexel University (Nyirjesy), Downtown Women’s Health Care (Waldbaum), the University of Alabama (Schwebke), and Tidewater Clinical Research, Inc. (Morgan), by Symbiomix Therapeutics, LLC, Baltimore, MD
Nunez*,2005* [16]	- Parallel design - Two arm - Phase III - Country: Manuel Noriega Trigo Hospital, Maracaibo, Venezuela - Follow-up period: one week - Unit of randomization: participant - Analysis unit: participant	- Enrolled: 76 - Randomization: 1 g single oral dose Secnidazole: 44 patients, 2 g single oral dose Secnidazole: 32 patients - Age: Mean (SD): Secnidazole 2 g: 39.4 (9.9); Senidazole 1 g: 41.1 (11.6) - Number of BV episodes in past 12 months: Not reported - Baseline nugent score: Not reported	1. single oral dose of Secnidazole 1 g 2. single oral dose of Secnidazole 2 g	- Primary: clinical cure: defined as an absence of the characteristic symptoms of BV (bad odor and a grossly abnormal discharge), and at least 2 of the following: vaginal pH less than 4.5, no fish odor on addition of KOH, and no G. vaginalis or clue cells on wet-mount examination. - Secondary: cytologic cure: was defined as an absence of G. vaginalis on a Pap smear	- A priori sample size estimation: No - Trial conduction date: Not reported - Sponsor: Not reported
Bohbot, 2010 [13]	- Parallel design - Two arm - Phase III - Country: France: Multicenter (27 centers) - Follow-up period: Assessed after 14 days and after 28 days - Unit of randomization: participant - Analysis unit: participant	- Number enrolled: 577 - Randomized: 1) metronidazole group: 237 in modified intention to treat analysis group and 2) Secnidazole group: 243 in modified intention to treat analysis group - Age: mean age: 36 years in both groups - Number of BV episodes in past 12 months: Approximately 28% of patients (secnidazole: 27.2%; metronidazole: 28.6%) had experienced at least one episode of BV during the two years preceding inclusion. - Baseline nugent score: Not reported	1. Intervention: 500 mg metronidazole twice per day for 7 days 2. Control: single oral dose of Secnidazole 2 g	*- Primary outcomes:* 1) Clinical cure: was defined as the normalisation of the three Amsel criteria and 2) bacteriological cure: was defined as a Nugent score lower or equal than three *- Secondary outcomes:* *therapeutic success at D14, clinical cure at D14 and D28, bacteriological cure at D14 and D28, mean time to symptom disappearance, and safety* *-* Safety assessments*:* *Safety was assessed on the basis of adverse events reported*	- Trial registration number: Not reported - Trial conduction date: between March 2007 and July 2008
Saracoglu, 1998 [22]	- Parallel design - 8 arms - Phase III Country: Turky: Ankara Numune Hospital Obstetrics and Gynecology Outpatient Clinic - Follow-up period: Assessed during first week and after 30–40 days “We called the patients to inform us about their symptoms after the first week and all the patients visited the clinic within 30–40 days for evaluation of the clinical and laboratory results” - Unit of randomization: participant - Analysis unit: participant	- Enrolled: 152 - Randomized: 1) Oral ornidazole: 34 pts. 2) Vaginal ornidazole: 21 3) Oral +vaginal ornidazole: 14 4) Oral secnidazole: 29 5) Oral secnidazole + vaginal ornidazole: 10 6) Oral secnidazole + vaginal metronidazole: 11 7) Oral ornidazole + vaginal metronidazole: 10 8) Vaginal metronidazole: 23 - Age: The ages of the patients in the treatment groups ranged between 19 and 45 with no statistical difference in between. - Number of BV episodes in past 12 months: Not reported - Baseline nugent score: Not reported	1. oral single dose 2 g Secnidazole 2. “oral secnidazole 2 g in a single dose and vaginal ornidazole 500 mg/day for 5 days” 3. “oral secnidazole 2 g in a single dose and vaginal metronidazole 2X 500 mg for 7 days” 4. “oral ornidazole 2X500 mg/day for 5 days and vaginal metronidazole 2X500 mg/day for 7 days;” 5. “vaginal metronidazole 2X500 mgrday for 7 days” 6. oral ornidazole 2 X 500 mg /day for 5 days 7. vaginal ornidazole 500 mg/day for 5 days 8. Oral + vaginal ornidazole for 5 days	Clinical cure: Absence of symptoms, vaginal discharge resulting from bacterial vaginosis and clue cells was accepted as cure.	- Trial registration number: Not reported - A priori sample size estimation: no - Trial conduction date: between January and May 1996 - Sponsor: Not reported - Role of sponsor: No reported
Schwebke, 2017 [20]	- Parallel design - Two arms - Phase III - Country: USA: Multicenter (21 center in USA). - Follow-up period: 21 to 30 days - Unit of randomization: participant - Analysis unit: participant	- Enrolled: 164 - Randomized: 1) 107 ptients to Secnidazole 2 g, 2) 57 patients to placebo group - Age: mean (SD): 1) Secnidazole group: 32 (8.7) and 2) placebo group: 30 (7.6) - Number of BV episodes in past 12 months: Mean (SD): 1) Secnidazole group: 3 (2.4) and 2) Placebo: 3 (2.6) - Number of BV episodes in past 12 months, n (%):1) ≤3: Secnidazole 2 g:83 (77.6), Placebo: 43 (75.4) . 2) ≥4: Secnidazole 2 g: 24 (22.4); Placebo: 14 (24.6) - Baseline Nugent score: mean (SD): 1) Secnidazole gourp: 8 (1.8); Placebo gourp: 9 (1.3)	1. oral single dose 2 g Secnidazole 2. placebo	- Primary: “Proportion of clinical outcome responders (CORs): Normal discharge, negative KOH whiff test, and clue cells < 20% at TOC/EOS visit (study days 21–30) - Secondary: “An alternate definition of responder defined as: Normal discharge after treatment or abnormal discharge that is inconsistent with BV; negative KOH whiff test; clue cells < 20% assessed at the interim visit (study days 7–14) and TOC/EOS (study days 21–30) - Safety: “Rates of adverse events (AEs), serious AEs, vital signs, physical examination findings, and laboratory test results	- Trial registration number: NCT02418845 - A priori sample size estimation: yes - Trial conduction date: April 16, 2015 to March 30, 2016 - Sponsor: The study was funded by Symbiomix Therapeutics. Dr. Schwebke received grant
Thulkar, 2012 [21]	- Parallel design - Four arms - Phase III - Country: Department of Obstetrics and Gynecology, All India Institute of Medical Sciences, New Delhi, India - Follow-up period: four weeks - Unit of randomization: participant - Analysis unit: participant	- Enrolled: 344 - Randomized: 86 patients to each group -Age: mean (SD): 27.9 ± 4.5 years, with a range of 20–40 years - Number of BV episodes in past 12 months: Not reported - Baseline Nugent score: Not reported	1. oral single dose of Secnidazole (2 g) 2. oral single dose of Metronidazole (2 g) 3. oral single dose of Tinidazole (2 g) 4. oral single dose of Ornidazole (1.5 g)	- Primary: Cure rate using Amsel criteria: Complete cure was considered when none of the four criteria were present. Improvement in the disease was considered when only one criterion was present. Partial cure was labelled when two criteria were present, and failure of treatment was labelled when three or four criteria were present.” - Secondary: effect on vaginal flora, and recurrence rate	- Trial registration number: This clinical trial was registered at the Clinical Trials Registry-India (CTRI; Reg. No: 2009–001093) - A priori sample size estimation: yes - Trial conduction date: from December 2008 to November 2009

We excluded four studies, because we were not able to find the full texts of those studies or they did not have a control group [23–26].

### Risk of bias in included studies

Four out of six trials were rated as having a low risk of selection bias as they used appropriate random sequence generation method. However, only one trial had low risk for allocation concealment bias. One trial stated that the investigators were not blinded to the treatment. The remaining four trials reported no information for allocation concealment, thus they were rated having an unclear risk for selection bias (Fig. 1). For assessment the risk of bias and quality of studies, we used Grading of Recommendations, Assessment, Development and Evaluation (GRADEpro, Table 2).

**Table 2:** Summary of findings table for presenting risks and quality of evidence about recruited studies

One gr Secnidazole compared to placebo for bacterial vaginosis Bibliography: secnidazole for bacterial vaginosis. Cochrane Database of Systematic Reviews [Year], Issue [Issue].											
Certainty assessment							Summary of findings				
№ of participants (studies) Follow-up	Risk of bias	Inconsistency	Indirectness	Imprecision	Publication bias	Overall certainty of evidence	Study event rates (%)		Relative effect (95% CI)	Anticipated absolute effects	
							With placebo	With 1 g Secnidazole		Risk with placebo	Risk difference with 1 g Secnidazole
clinical cure rate											
126 (1 RCT)	not serious	not serious	not serious	not serious	none	⨁⨁⨁⨁ HIGH	11/62 (17.7%)	33/64 (51.6%)	RR 2.92* (1.64 to 5.19)	177 per 1000	**341 more per 1000** (from 114 more to 743 more)
clinical cure rate - Clinical cure of pts. with 3 or less episodes of BV in the last year											
87 (1 RCT)	not serious	not serious	not serious	not serious	none	⨁⨁⨁⨁ HIGH	10/43 (23.3%)	26/44 (59.1%)	RR 2.54* (1.40 to 4.61)	233 per 1000	**358 more per 1000** (from 93 more to 840 more)
clinical cure rate - Clinical cure of pts. with 4 or more episodes of BV in the last year											
39 (1 RCT)	not serious	not serious	not serious	very serious ^a^	none	⨁⨁ ◯◯ LOW	1/19 (5.3%)	7/20 (35.0%)	**RR 6.65** (0.90 to 49.09)	53 per 1000	**297 more per 1000** (from 5 fewer to 1000 more)
Microbiologic cure rate											
126 (1 RCT)	not serious	not serious	not serious	not serious	none	⨁⨁⨁⨁ HIGH	4/62 (6.5%)	15/64 (23.4%)	RR 3.35* (1.26 to 8.95)	65 per 1000	**152 more per 1000** (from 17 more to 513 more)
Microbiologic cure rate - Microbiologic cure of pts. with 3 or less episodes of BV in the last year											
87 (1 RCT)	not serious	not serious	not serious	not serious	none	⨁⨁⨁⨁ HIGH	4/43 (9.3%)	13/44 (29.5%)	RR 3.18* (1.12 to 8.98)	93 per 1000	**203 more per 1000** (from 11 more to 742 more)
Therapeutic cure rate											
126 (1 RCT)	not serious	not serious	not serious	not serious	none	⨁⨁⨁⨁ HIGH	4/62 (6.5%)	14/64 (21.9%)	RR 3.14* (1.17 to 8.42)	65 per 1000	**138 more per 1000** (from 11 more to 479 more)
Therapeutic cure rate - Therapeutic cure of pts. with 3 or less episodes of BV in the last year											
87 (1 RCT)	not serious	not serious	not serious	not serious	none	⨁⨁⨁⨁ HIGH	4/43 (9.3%)	13/44 (29.5%)	RR 3.18* (1.12 to 8.98)	93 per 1000	**203 more per 1000** (from 11 more to 742 more)
Therapeutic cure rate - Therapeutic cure of pts. with 4 or more episodes of BV in the last year											

* significant (*p* <0.05)

*CI* Confidence interval, *RR* Risk ratio

Explanations

^a^A very wide confidence interval

^b^Large confidence interval

Bold data are significant

### Effects of interventions

#### 2 g secnidazole versus placebo

**Fig. 3:**
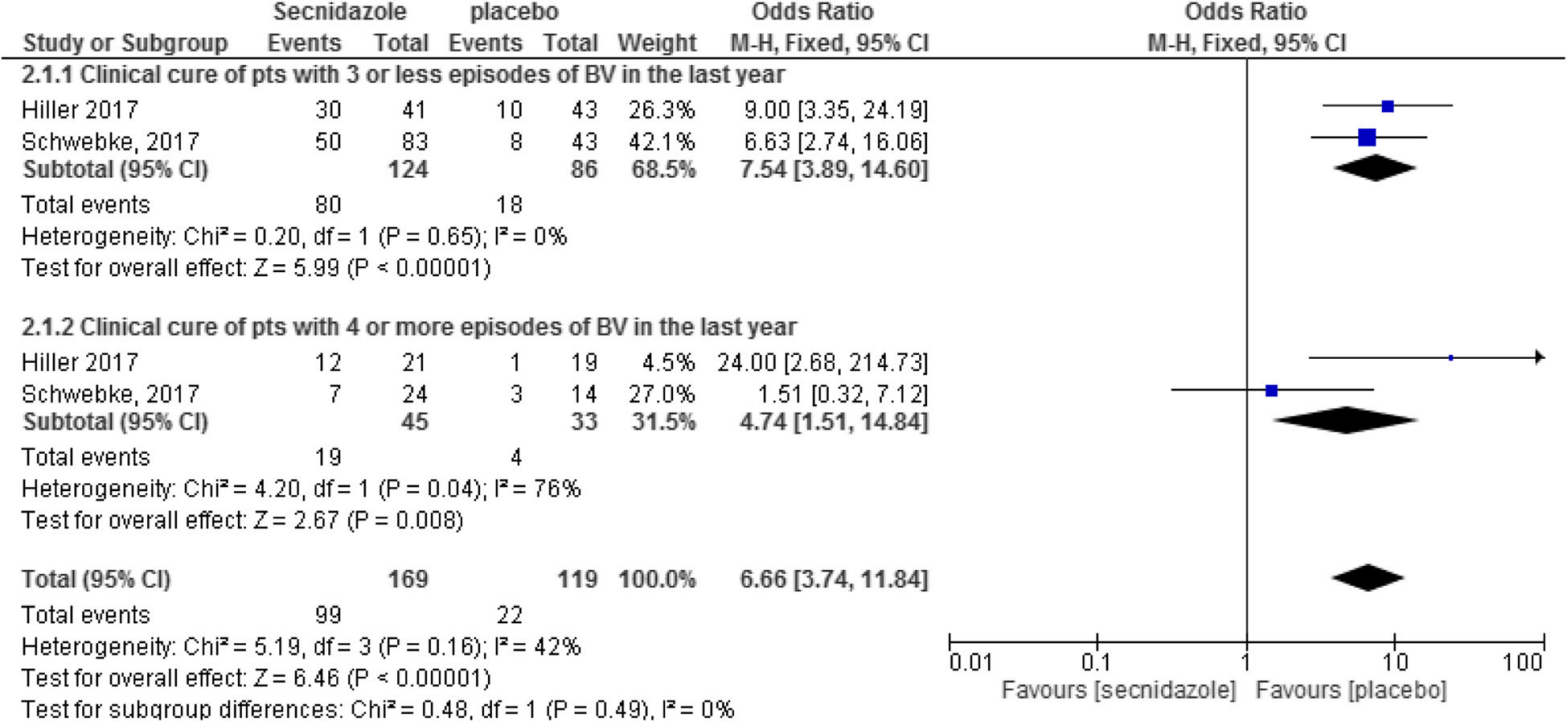
Forest plot of clinical cure of patients with three or four episodes of BV in the last year

The effect of intervention in terms of clinical cure of patients with three or less episodes of BV in the last year is illustrated in Fig. 3. Two studies [17, 20] with total participants of 210 were recruited in the meta-analysis. As evident from this figure, treatment with 2 g secnidazole could significantly reduce the risk of BV in patients with three or less episodes of BV in the last year 7.54 (95% CI: 3.89–14.60, *p* < 0.00001) or in patients with four or more episodes of BV in the last year (OR: 4.74, 95% CI: 1.51–14.84, *p* = 0.0.008) (*n* = 78). The level of heterogeneity was high (I^2^ = 76%) in the later analysis and did not change using random effect model. Because only two studies were in the meta-analysis, the sensitivity analysis was not applicable (Fig. 3) [17, 20].

The microbiologic cure rate of 2 g secnidazole versus placebo was assessed in one study [17] (*n* = 124). Results showed that 2 g secnidazole could significantly increase the microbiologic cure rate in women with 3 or less episodes of BV in the last year (OR: 7.36, 95% CI: 2.30–25.33, *p* = 0.0009) but not in the women with 4 or more episodes of BV in the last year (OR: 20.17, 95% CI: 1.06–382.45.78, *p* = 0.05). Also the Hiller’s study (n = 124) showed that therapeutic cure rate of 2 g secnidazole versus placebo was 9.34 (OR: 9.34, 95% CI: 3.10–28.11, *p* = 0.0001) [17].

#### 2 g versus 1 g secnidazole

**Fig. 4:**
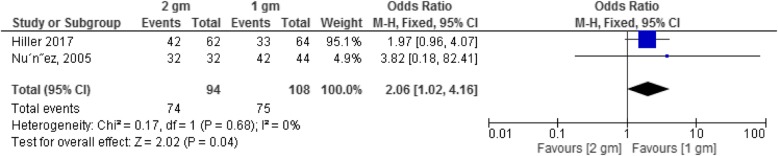
Forest plot of clinical cure of BV in 2 g versus 1 g secnidazole

The comparison of effect of 2 g versus 1 g secnidazole is illustrated in Fig. 4 (*n* = 202). As shown in this figure the clinical cure rate of 2 g secnidazole was significantly more than that of 1 g secnidazole (OR: 2.06, 95% CI: 1.02–4.16, *p* = 0.04) [16, 17].

Two studies comprising 202 women used 2 g versus 1 g secnidazole for assessing the microbiological effect [16, 17]. There was a significant difference between 2 g versus 1 g secnidazole regarding microbiological effect (OR: 2.21, 95% CI: 1.07–4.60, *P* = 0.03). The therapeutic cure rate in patients who received 2 g secnidazole was significantly more than that in 1 g secnidazole (OR: 8.07, 95% CI: 2.81–123.17, *p* = 0.0001) (*n* = 125) [17].

#### 1 g secnidazole versus placebo

**Fig. 5:**
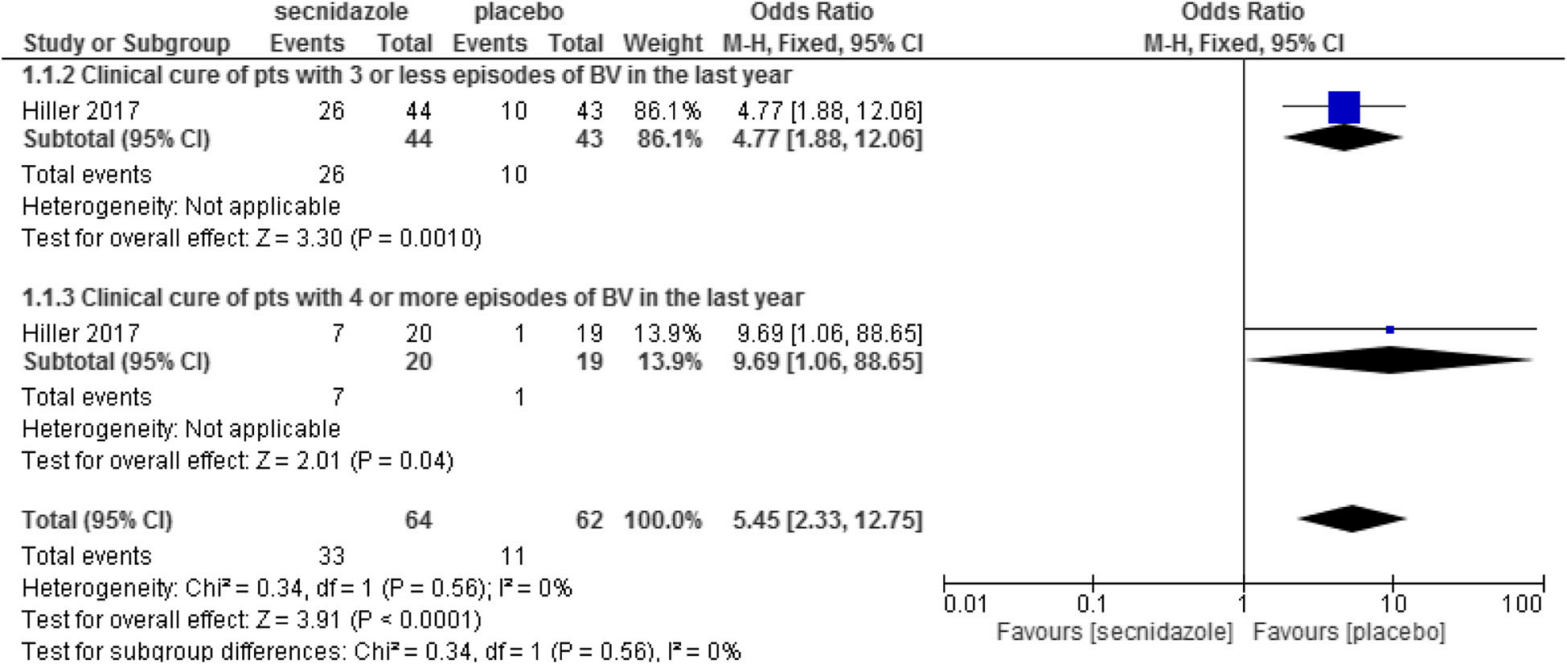
Clinical cure rate of 1 g secnidazole versus placebo

The clinical cure rate of 1 g secnidazole versus placebo was evaluated in 126 women and results showed that secnidazole was significantly more effective than placebo (OR: 5.45, 95% CI: 2.33–12.75, p = 0.0001) (Fig. 5) [17].

The microbiologic cure and the therapeutic cure rate of 1 g secnidazole was significantly more than that in the placebo group (OR: 4.25, 95%CI: 1.37–13.18, *p* = 0.01) and (OR: 3.14, 95% CI: 1.17–8.42, *p* = 0.02) respectively [17].

#### Secnidazole versus metronidazole

One study [13] recruited 480 women to compare the effect of 2 g secnidazole versus metronidazole (500 mg bid for 5 days). Results showed that there was no significant difference between two groups in terms of clinical cure (OR: 0.87, 95% CI: 0.56–1.34, *P* = 0.53). Also the effect of 2 g oral secnidazole versus 2 g secnidazole plus vaginal metronidazole was compared in one study with 40 participants and the results showed that there was no significant difference between two groups (OR: 0.11, 95% CI: 0.01–2.08, *p* = 0.14) [22]. Another study with 172 participants for comparing the effect of 2 g oral secnidazole versus 2 g single dose oral metronidazole showed no significant difference between two groups regarding cure rate after one week using Amsel criteria (OR:1.28, 95% CI: 0.48–3.42, *p* = 0.62), or after 4 weeks (OR: 1.15, 95% CI: 0.55–2.40, *p* = 0.71) [21].

One study recruiting 39 women evaluated the effect of 2 g secnidazole versus 2 g secnidazole plus vaginal ornidazole and results showed no significant difference between two groups (OR: 0.29, 95% CI: 0.03–2.69, *p* = 0.28) [22]. Also the effect of 2 g oral secnidazole versus 2 g secnidazole plus vaginal metronidazole was compared in one study with 40 participants and the results showed that there was no significant difference between two groups (OR: 0.11, 95% CI: 0.01–2.08, p = 0.14).

#### Adverse effect

The adverse effects that reported in studies that 2 g secnidazole was used versus placebo were yeast infection, vulvovaginal pruritus, dyspareunia, nausea, increase the level of hepatic enzymes such as ALT and AST and headache. There was no significant difference between the two groups in any of the studies regarding adverse effects in 2 g secnidazole versus placebo (*n* = 233) (OR: 2.24, 95% CI: 0.85–5.94, *P* = 0.10) or versus 1 g secnidazole (*n* = 219) (OR: 1.25, 95% CI: 0.65–2.41, *P* = 0.50) [17, 20].

## Discussion

This systematic review designed to evaluate the effect of secnidazole on bacterial vaginosis in childbearing women.

BV is one of the most prevalent vaginitis in the United States and almost 30% of childbearing aged women are affected with this infection [27]. BV has some consequences such as; endometritis, postpartum fever, cellulitis in the hysterectomy cuff and infection after abortion [28] and also is a risk factor for acquisition of HIV and herpes simplex virus type 2 and other sexually transmitted diseases [29].

The main medication for treatment of BV is metronidazole that has 90% effectiveness, but the recurrence rate is high [30]. Secnidazole is a next-generation of 5-nitroimidazole that has already approved in Europe and Asia as a single dose of 2 g for BV. This medication also was approved to use in the USA in 2004 [31]. The long half- life of secnidazole (17–28.8 h) makes it possible for a single dose to be effective [32]. The food and drug administration (FDA) of the United States recently approved the single 2 g dose of secnidazole for the treatment of BV according to two randomized controlled trials that conducted in the United States [33].

The results of this systematic review showed that 1 g secnidazole could significantly improve the clinical cure rate in women with BV compared to placebo, but the microbiological cure rate was not significantly different from placebo in women with 4 or more episodes of BV in the last year.

Our results also revealed that 2 g secnidazole significantly could treat BV in women with three or less or four and more episodes of BV in the last year in compare to placebo. Further, our results showed that 2 g secnidazole was significantly more effective in terms of clinical cure rate and microbiological impact than that of 1 g secnidazole.

We found only one study that compared the effect of 2 g secnidazole with metronidazole (500 mg bid for 5 days) or 2 g single dose of metronidazole. The results revealed no significant difference regarding clinical cure rate in both methods.

The diagnosis of BV is according to the Amsel criteria (at least three criteria should be present) including; homogenous grayish-white vaginal discharge, vaginal pH > 4.5, positive whiff test (fish odor with adding a drop of 10% potassium hydroxide to the vaginal discharge) and presence of more than 20% clue cells in the wet smear of vaginal discharge [34]. The clinical cure of BV are including; a negative test for amino odor after adding 10% potassium hydroxide solution to vaginal discharge, the number of clue cells less than 20% and pH of vaginal discharge < 4.5 [35]. In all studies recruited in this systematic review the Amsel criteria was used for clinical cure rate of BV.

### Strengths and limitations of the study

This is the first time that we evaluated the effect of secnidazole on BV in a systematic review study. In this systematic review we only found six studies and the meta-analysis was only possible in some cases. High level of heterogeneity was observed in some studies that meta-analyses were performed. Because in most cases there were only two studies in the meta-analysis, the sensitivity analysis was not possible. Therefore the results should be considered with caution.

## Conclusion

This systematic review showed that 2 g and 1 g secnidazole were better than placebo, however, 2 g secnidazole was more effective than 1 g. Secnidazole 2 g was not different from metronidazole (500 mg bid for 5 days), or from secnidazole plus vaginal metronidazole, or 2 g single dose of oral metronidazole or from 2 g secnidazole plus vaginal ornidazole. Secnidazole can be considered as an alternative to the treatment of BV for women who have experienced adverse effects or had a recurrence with current medications of BV.

## Supplementary information

**Additional file 1.** Searches strategies.

## Data Availability

Data sharing is not applicable for this study. There is no datasets were used for this study.

## Abbreviations

Bid: twice (two times) a day
BV: Bacterial vaginosis
RevMan: Review Manager
