# Supplementary material for: Secnidazole for treatment of bacterial vaginosis: a systematic review
Source: BMC Womens Health. 2019 Oct 21;19:121. doi: 10.1186/s12905-019-0822-2 (PMC6802328; doi:10.1186/s12905-019-0822-2)
Supplement: Supplementary file 1 — Additional file 1. Searches strategies. [file 12905_2019_822_MOESM1_ESM.docx]

**Supplemental material # 1**

**Searches strategies**

|  |  |
| --- | --- |
| **MEDLINE**  PubMed  October 28^th^,2018 | ("secnidazole"[Supplementary Concept] OR "secnidazole"[Title/abstract] OR Secnil[Title/abstract] OR Solosec[Title/abstract] OR SYM-1219[Title/abstract] OR "nitroimidazoles"[MeSH Terms] OR "nitroimidazoles"[Title/abstract] OR "nitroimidazole"[Title/abstract]) AND  ((("vaginosis, bacterial"[MeSH Terms] OR ("vaginosis"[Title/abstract] AND "bacterial"[Title/abstract]) OR "bacterial vaginosis"[Title/abstract] OR ("bacterial"[Title/abstract] AND "vaginosis"[Title/abstract])) OR (("vaginitis"[MeSH Terms] OR "vaginitis"[Title/abstract]) AND bacterial[Title/abstract]))) |
| **Scopus**  October 28th,2018 | ( ( TITLE-ABS-KEY ( secnidazole ) OR TITLE-ABS-KEY ( secnil ) OR TITLE-ABS-KEY ( solosec ) OR TITLE-ABS-KEY ( sym-1219 ) OR TITLE-ABS-KEY ( nitroimidazoles ) OR TITLE-ABS-KEY ( nitroimidazole ) ) AND ( TITLE-ABS-KEY ( bacterial AND vaginosis ) OR TITLE-ABS-KEY ( bacterial AND vaginoses ) OR TITLE-ABS-KEY ( "vaginosis" AND "bacterial" ) OR TITLE-ABS-KEY ( vaginitis AND bacterial ) ) ) |
| **Cochrane Central Register of Controlled Trials :**  The Cochrane Library  October 28th,2018 | #1 (secnidazole):ti,ab,kw OR (Secnil):ti,ab,kw OR (Solosec):ti,ab,kw OR (SYM-1219):ti,ab,kw OR (nitroimidazoles):ti,ab,kw  word variations have been searched 402  #2 MeSH descriptor: [Nitroimidazoles] explode all trees 2513  #3 (nitroimidazole):ti,ab,kw (Word variations have been searched) 341  #4 MeSH descriptor: [Vaginosis, Bacterial] explode all trees 349  #5 ("bacterial vaginoses"):ti,ab,kw OR ("bacterial vaginosis"):ti,ab,kw (Word variations have been searched) 618  #6 (vaginitis):ti,ab,kw AND (bacterial):ti,ab,kw (Word variations have been searched) 952  #7 #1 or #2 or #3 2616  #8 #4 or #5 or #6 1052  #9 #7 and #8 221 |
| **Web of science**  All databases  (1864-2018**)**  October 28th,2018 | #1 TS= ((secnidazole) OR (Secnil) OR (Solosec) OR (SYM-1219) OR (nitroimidazoles)) 5668  #2 TS=(nitroimidazole) 3803  #3 TS= (("bacterial vaginoses") OR ("bacterial vaginosis")) 7110    #4 TS= ((vaginitis) AND (bacterial)) 4495  #5 #1 or #2 7760  #6 #3 or #4 9447  #7 #5 and #6 109 |
